# Peer-Delivery of a Gender-Specific Smoking Cessation Intervention for Women Living in Disadvantaged Communities in Ireland We Can Quit2 (WCQ2)—A Pilot Cluster Randomized Controlled Trial

**DOI:** 10.1093/ntr/ntab242

**Published:** 2021-11-20

**Authors:** Catherine B Hayes, Jenny Patterson, Stefania Castello, Emma Burke, Nicola O’Connell, Catherine D Darker, Linda Bauld, Joanne Vance, Aurelia Ciblis, Fiona Dobbie, Kirsty Loudon, Declan Devane, Nadine Dougall

**Affiliations:** 1 Public Health and Primary Care, Institute of Population Health, School of Medicine, Trinity College Dublin, Dublin, Ireland; 2 School of Health and Social Care, Edinburgh Napier University, Edinburgh, Scotland; 3 Usher Institute and SPECTRUM Consortium, College of Medicine and Veterinary Science, University of Edinburgh, Edinburgh, Scotland; 4 Irish Cancer Society, Dublin, Ireland; 5 Freelance Researcher, Dublin, Ireland; 6 Usher Institute, College of Medicine and Veterinary Science, University of Edinburgh, Edinburgh, Scotland; 7 Freelance Researcher, Edinburgh, Scotland; 8 HRB Trials Methodology Research Network, School of Nursing & Midwifery, NUI Galway, Galway, Ireland

## Abstract

**Introduction:**

We Can Quit” (WCQ) is community-based stop-smoking program delivered by trained community facilitators, based on the socio-ecological framework and developed using a Community-based Participatory Research approach, targeting women living in socioeconomically disadvantaged (SED) areas of Ireland.

**Aims and Methods:**

The We Can Quit2 (WCQ2) pilot trial assessed the feasibility of WCQ. A pragmatic cluster randomized controlled trial with a process evaluation WCQ2, was conducted in four matched pairs of SED districts (8–10 000 women per district). Districts were independently randomized to WCQ (group support + nicotine replacement therapy), or to individual support delivered by health professionals. Participants were adult women smokers interested in quitting, who were living or working in trial districts. Recruitment of districts and 194 women in four waves (49 women per wave); retention at 12 weeks and 6 months; fidelity to intervention delivery and acceptability of trial-related processes were assessed. Validated smoking abstinence at 12-week and 6-month post-intervention was recorded, missing data assumed as continued smoking.

**Results:**

Eight districts were recruited. 125/188 (66.5%) eligible women consented. The 49 women target was reached in wave4. Retention at 12 weeks was (Intervention [I]: 55.4%; Control [C]: 51.7%), at 6 months (I: 47.7%; C: 46.7%). Smoking abstinence at 12 weeks was (I: 23.1%, [95% CI: 14.5 to 34.7]; C: 13%, [95% CI: 6.9 to 24.1]). 83.8% of session activities were delivered. Trial-related processes were acceptable to facilitators. Low literacy was highlighted as a barrier for participants’ acceptability.

**Conclusions:**

WCQ was feasible to deliver by trained facilitators and indicated a positive direction in abstinence rates. Low literacy will need to be addressed in a future trial design.

**Implications:**

This pilot trial showed that a stop-smoking intervention tailored to a group of women smokers living in SED areas which was delivered by trained local women within their local communities was feasible. Furthermore, although not formally compared, more WCQ women were abstinent from smoking at the end of treatment. The results are relevant to enhance the design of a fully powered effectiveness trial, and provide important evidence on the barriers to deliver a tailored smoking cessation service to SED women smokers in Ireland.

## Introduction

Tobacco use is the leading cause of preventable death globally.^1^ It is a critical factor in the etiology of chronic diseases, for example, cardiovascular disease and at least 12 types of cancer.^2^ It is linked causally to lung cancer,^3^ the leading cause of cancer death in men and women in high-income countries.^4^ The health consequences of smoking are disproportionately borne by socioeconomically disadvantaged (SED) populations.^5^ SED groups are more exposed to daily stressors and have fewer material resources to control the sources of stress.^6^ These factors have been associated with higher smoking prevalence and tobacco exposure.^1,7^ As a consequence, SED populations develop higher rates of illness and death, which increase health disparities.^1,8^ Women are more likely to smoke to cope with negative emotions and stressful situations, experience more difficulties in quitting, and are more likely to relapse.^9,10^ SED contributes to higher tobacco use among adult women and influences their smoking status in terms of smoking initiation, persistence, consumption, and cessation.^11^ This pattern of smoking in women is likely to affect subsequent generations via role-modeling or exposure to second-hand smoke.^12^

The World Health Organization Framework Convention on Tobacco Control recommends that tobacco control measures target SED groups.^5^ It has highlighted the need for approaches tailored to gender when developing tobacco control policies in the light of increasing lung cancer rates in women,^5^ which have surpassed breast cancer rates in many countries including Ireland.^13,14^ Lung cancer is the leading cause of cancer death in Irish women,^13^ and represents the highest incidence rates in Europe. Similar to other Western countries,^1^ incidence and mortality from lung cancer are highest in SED populations.^15^ A systematic review carried out by our team^16^ (manuscript under review), revealed seven randomized controlled trials (RCTs) of stop-smoking interventions in women from SED areas.^17–23^ All studies delivered individual interventions, except one which included group support.^17^ More evidence is needed of effectiveness of smoking cessation interventions tailored not only to individual aspects of tobacco use by vulnerable women but also to the socioeconomic circumstances of their lives.^11,24^

We Can Quit (WCQ) is a community-based smoking cessation program based on the socio-ecological framework^25^ and developed using a Community-based Participatory Research approach.^26^ The WCQ intervention comprises group-based behavioral support delivered by trained community facilitators (CFs). It was specifically designed for women living in SED areas in Ireland.^27^ Intervention development is fully described elsewhere.^27,28^ It was evaluated in a small, single-arm feasibility study, with 74.3% retention and 46% cessation rates (self-report and Carbon Monoxide breath test) at end of program.^27^ The We Can Quit2 (WCQ2) pilot trial reported here builds on the previous feasibility study.

The overarching aim of the WCQ2 trial was to test the feasibility of conducting a trial of delivery of the WCQ smoking cessation intervention to SED women smokers in preparation for a future definitive trial (DT) of effectiveness. Objectives addressed in this manuscript which are published in the trial protocol,^28^ were (1) to determine the feasibility and acceptability of trial processes including recruitment, randomization, and data collection; (2) to assess data completion rates for the main outcome measures including smoking abstinence at 12 weeks (end of program) and 6 months (longer-term outcome); and (3) to inform sample size estimates including an estimate of the intra-cluster correlation coefficient to account for the effect of “clustering” in design and analysis. The stated secondary objective *to test the robustness of trial design with respect to context for delivery of the intervention, implementation processes, and key mechanisms of impact,* includes only principal relevant findings in this manuscript. A further stated objective *strategies to optimize recruitment and dissemination of findings to trial stakeholders* is not addressed due to space constraints.

## Methods

### Design

WCQ2 was a pragmatic two-arm, parallel-group pilot cluster RCT conducted in selected SED districts of Dublin and Cork in Ireland between September 2017 and September 2019. Eight districts (four cluster pairs) of approximately 8–10,000 women per district were identified according to cluster eligibility criteria.^28^ A mixed-methods process evaluation was also conducted. The University of Dublin, Trinity College School of Medicine Research Ethics Committee provided ethical approval (reference 20170404). The trial protocol was submitted for publication prior to completion of data collection (October 2018).^28^ The trial was retrospectively registered on 24/9/2018 (controlled trials ISRCTN74721694).

### Participants

Participants were recruited from the general population of women living in selected SED districts. A target of 50% of women eligible for general medical services (GMS) was set (national estimated average 43%).^29^ The GMS scheme in Ireland provides access to health services for persons for whom acquiring such services would present undue hardship.^29^ Eligible women were aged 18 or over who resided or worked in the trial districts who were fluent English speakers; self-reported as daily smokers in the previous 7 days and interested in quitting. Women using Nicotine Replacement Therapy (NRT), e-cigarettes, or prescribed bupropion/varenicline at time of recruitment, were eligible. Excluded women were those pregnant or actively planning a pregnancy, not capable of providing informed consent, or who were previously enrolled in another smoking cessation study.

The Irish Cancer Society (ICS) established Local (Area) Advisory Groups (LAGs), which included representation from the Health Service Executive (HSE), ICS, and community groups to oversee trial planning in each cluster pair.^28^

Recruitment occurred in four sequential time periods (waves) according to the availability of trained CFs. The recruitment plan estimated a 12-week period to engage relevant community and primary care stakeholders prior to active recruitment. Stakeholders and LAG members promoted the study with assistance from the research team via key contacts, local service users, social media, and community events, to identify suitable participants.

Participant recruitment was estimated to take 8 weeks per wave. Interested women self-registered or were assisted to register on the trial website and were screened against eligibility criteria by phone. Eligible women received a participant information leaflet and consent form at least 24 h before providing written informed consent at a community venue.

### Randomization and blinding

The Wellcome Trust Clinical Research Facility independently randomized each matched district pair to receive the WCQ intervention or HSE usual care (control arm) in a 1:1 ratio using a secure web-based program. Once recruitment had closed for each wave, the allocation code was revealed to the research team who informed program delivery personnel of their allocated district, who in turn informed participants of their allocation. Trial Statisticians were blinded to group allocation until analysis was completed.

### Procedure

#### Intervention

Participants randomized to the intervention arm received the WCQ program. Core components of WCQ were: (1) a face-to-face group-based behavioral support program delivered weekly in 90-minute sessions over 12 weeks; (2) optional access to combination NRT, available free of charge to all participants and dispensed by community pharmacists; (3) intervention delivered by trained CFs to women in their local community setting, for example, resource center.^28^

The CFs who delivered the program were community development/health staff who had worked with SED women and may have had experience in delivering stop-smoking support for ICS. Suitable CFs were identified by the LAGs for training. Their training program complied with all the HSE and National Standard guidelines for smoking cessation, and additional wrap-round elements as co-designed with the community in the previous development study. It incorporated the National Women Council Ireland training guidelines for gender mainstreaming. The focus was to explore the role of gender and other social determinants of health on smoking and quitting; building a holistic, women-centerd and empowering health and wellbeing approach and addressing relationships between smoking and other lifestyle factors.^27,28^ The training was co-delivered by HSE and ICS’s prevention team.

CFs received an intervention manual and additional material tailored to women with low literacy. They delivered the sessions in pairs, in an empathetic and listening environment. The recommended number of participants in each session was 8–15. CFs delivered specific activities and information on pre-defined topics, involving peer-discussion, feedback on activities of the previous week, and home exercises. See Supplementary File S1 for details of session contents. Sessions 7–12 included optional activities chosen by each group of women at session 6, which were tailored to their preferences and needs. These activities were focused on increasing self-efficacy, providing peer-support by sharing experiences, and celebrating achievements with family, friends, and the local community.

GMS participants required a prescription from their GP to obtain NRT products; non-GMS cardholders collected directly from a pre-designated community pharmacist who also provided specific advice on NRT use.

#### Control Arm

Participants randomized to the control arm received an individual smoking cessation program offered by the HSE.^30^ This comprised, on average, 6–7 individual contacts delivered by a Smoking Cessation Officer in a primary care center or hospital outpatient clinic. Key program components include reinforcing motivation to quit, building coping mechanisms, and providing information on tobacco addiction and withdrawal.^28^ Session one was delivered face-to-face and lasted between 30 and 45 min. Subsequent sessions may have been phone-based, vary in duration, and delivered according to client needs. Participants obtained NRT free of charge if they were eligible for GMS.

#### Data Collection

Questionnaires (Supplementary File S2) were administered by the researcher at baseline prior to randomization, post-intervention at 12 weeks (12w, end of program), and at 6 months (6m).

Baseline data assessed socio-demographic characteristics, smoking behavior, and physical and mental health status measured using the 12-item short-form survey questionnaire SF-12.^31^

Program delivery personnel recorded attendance. They made repeated attempts to contact participants between sessions, and to reach non-attenders. Reasons given by participants for dropout at each point were recorded using a question guide.

Follow-up questionnaires were administered between May 2018 and August 2019, and addressed smoking status, changes in smoking behaviors, NRT use, and changes in health status. Participants who self-reported smoking abstinence at both time points followed specific guidance to provide saliva samples for cotinine and anabasine analysis.^32^

The researcher contacted women up to three times to schedule flexible individual appointments to collect follow-up data in community locations, and sent SMS reminders before each meeting. Participants received a thank you payment (€20 shopping voucher) at each follow-up point for data collection.

Acceptability of trial-related processes was assessed through semi-structured face-to-face interviews with a purposive sample of 20 information-rich cases and all CFs.^28^ These were conducted (June 2018–May 2019) by an experienced qualitative researcher (E.B.) unknown to participants, using an interview guide (Supplementary File S3).^28^ Informed consent was obtained prior to interview. Participant interviews lasted 20–30 min. Interviews with CFs lasted an hour and were conducted jointly apart from wave3 when respondents requested separate interviews. Interviews were audio-recorded and observational field notes were completed.

The short four-item validated scales the Acceptability of Intervention Measure (AIM), Intervention Appropriateness Measure (IAM), and Feasibility of Intervention Measure (FIM)^33^ were self-administered by CFs to rate the delivery of WCQ. After each session, CFs registered the specific content delivered using a checklist to assess fidelity (Supplementary File S1).^28^

### Outcomes

The primary outcome was assessment of whether the recruitment target of eight districts (clusters) and 194 women was achievable within 18 months of program start. Other key outcomes were a retention target of 120 women (60 per arm) at 12w, retention rates at 6m follow-up, and engagement and attendance in each arm. Self-reported smoking abstinence corroborated by biochemical confirmation,^32^ and the percentage improvement in physical and mental health status were recorded at 12w and 6m. Key process evaluation outcomes were acceptability of trial-related processes by participants and CFs and fidelity to intervention delivery. Qualitative data on acceptability of the WCQ intervention will be reported separately (manuscript submitted).

### Data Analysis

Descriptive analysis for each group was conducted using an intention-to-treat model.^32^ Per-protocol (PP) analyses were also conducted. Dropouts prior to randomization were calculated from the difference between the number of eligible and consented participants, as a percentage of eligible participants. Recruitment was defined from the total number of consented women, as a percentage of those eligible. Retention was calculated from the number of women who completed data at 12w and 6m, as a percentage of consented participants.

Engagement was calculated from the number of participants who attended a session and set a quit date as a percentage of the total number of consented women in each arm. Median attendance (±IQR) was calculated from the number of sessions attended.

Saliva samples were analyzed in ABS Laboratories Ltd, Hertfordshire, UK. The proportion (95% CI) of participants with biochemically confirmed point-prevalence and continued smoking abstinence as per Russell Standard^32^ was reported for each arm at 12w and 6m. Missing data on smoking status were computed as if continued smoking.^32^ Mean (SD) percentage change in number of cigarettes smoked and the proportion (95% CI) of participants who improved their physical and mental health status were calculated between baseline and 12w, and baseline and 6m. Median attendance was calculated for participants who reported continued abstinence from smoking. The proportion of participants who accessed NRT, types used, and adherence were recorded. All quantitative data were analyzed using SPSS V23.0 (IBM Corp. Armonk, NY).

Data triangulation informed the acceptability of trial processes by intervention participants and CFs. Interview transcripts were analyzed following thematic analysis, and using NVivo (version 12, QSR International).^28^ AIM, IAM, and FIM items were rated on a 5-point Likert scale, with higher scores indicating greater acceptability, appropriateness, and feasibility. Ratings from each scale were summed and averaged across the four items. Number and percentage of self-reported activities delivered within each session were reported from fidelity checklists. Average scores from each wave provided an overall rate of activity completion across the WCQ program.

Criteria for progression to a DT were attainment of the recruitment and retention targets. Specific tools will be used to assist in the decision-making process on progression to a future DT^28^ (manuscript in preparation).

### Sample Size

We estimated a total sample size of 194 women (97 per arm), that is, 48–49 participants (24–25 per cluster) recruited into the study in four time periods (waves). Based on retention rates from the single-arm WCQ study,^27^ we estimated that 120 women (62%), 60 per trial arm would remain at 12w follow-up.^28^

## Results

### Primary Outcome

Recruitment of LAGs and clusters commenced in June 2017. Eight districts were recruited. Participant recruitment took place between January 2018 and February 2019 in four sequential waves.

**Figure 1.**
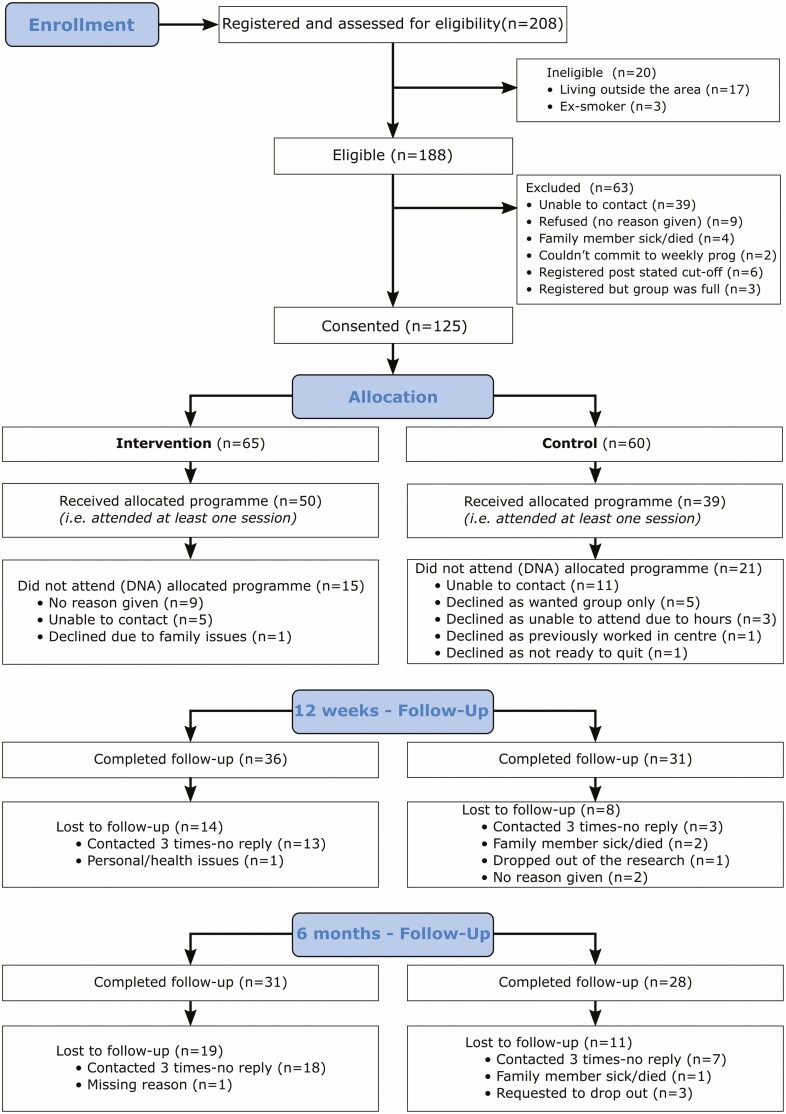
CONSORT diagram describing flow of participants through the study.

Registration took 8–12 weeks per wave (Figure 1). The eligibility rate was 90.4% (188/208). As 63 women (33.5%) dropped out before district randomization, 125/188 (66.5%) women consented and provided baseline data (recruitment rate), 64.4% of the recruitment target (125/194 women). The expected recruitment rate of 49 consented and enrolled women was achieved in wave4 (Table 1). Two groups were convened in this wave.

**Table 1.** Participant Recruitment Rates—Overall and by Wave

Wave	No. eligible	No. consented	Recruitment rate (%)	Target achieved (%)
1	40	23	56.1	46.9
2	45	30	66.6	61.2
3	29	23	79.3	46.9
4	74	49	65.3	100.0
Total	188	125	66.5	64.4

Sixty-five women were allocated to intervention (I) and 60 to control (C) arms. Overall, each trial arm was well-matched in terms of baseline socio-demographics and smoking variables (Supplementary Table 1) although some differences were noted. Most participants (97%) self-described as white Irish (not shown). The proportion of GMS participants exceeded 60%.

### Engagement, Attendance, and Retention

Thirty-six participants [I: *n* = 15; C: *n* = 21] did not attend either program or provide data post-baseline (Figure 1). Of these, 16 could not be contacted, while five control participants indicated a preference for WCQ. Therefore, the overall proportion of participants who engaged with smoking cessation services was 89/125 (71.2%, Table 2). The average number of sessions attended was less than half in each arm as per intention-to-treat analysis, this rose to 3/4 in the PP analysis.

**Table 2.** Engagement, Attendance, and Data completion rates

		Intervention	Control	Total
Engagement		*n* (%)	*n* (%)	*n* (%)
Overall		50/65 (76.9)	39/60 (65.0)	89 (71.2)
Wave 1		7/9(77.8)	6/14 (42.9)	13 (56.5)
Wave 2		13/20 (65.0)	9/10(90.0)	22 (73.3)
Wave 3		10/12 (83.3)	9/11(81.8)	19 (82.6)
Wave 4		20/24 (83.3)	15/25 (60.0)	35 (71.4)
Attendance^a^				
ITT *n* = 125	Mean (±SD; 95% CI)	5.2 (4.1; 4.2 to 6.2)	2.5 (2.4; 1.9 to 3.1)	
	Median (IQR)	5 (1-9)	2 (0-4)	
PP *n* = 89	Mean (±SD; 95% CI)	6.8 (3.3; 5.8 to 7.7)	3.9 (1.9; 3.2 to 4.5)	
	Median (IQR)	7.5 (4–10)	4 (2–5)	
Data completion rates		*n* (%)	*n* (%)	*n* (%)
ITT^b^ *n* = 125	Completed baseline	65 (100)	60 (100)	125 (100)
	Completed 12w follow-up	36 (55.4)	31 (51.7)	67 (53.6)
	Completed 6m follow-up	31 (47.7)	28 (46.7)	59 (47.2)
PP^c^ *n* = 89	Completed baseline	50 (100)	39 (100)	89 (100)
	Completed 12w follow-up	36 (72)	31 (79.5)	67 (75.3)
	Completed 6m follow-up	31 (62)	28 (71.8)	59 (66.3)

ITT = intention-to-treat analysis, including *n* = 65 in intervention and *n* = 60 in control arms; PP = per-protocol analysis, including *n* = 50 in intervention and *n* = 39 in control arms.

^a^Intervention: out of 12 sessions; Control: out of 6–7 sessions.

^b^Six participants (three per group) who did not complete data at 12w provided data at 6m.

^c^Six participants who completed 6m follow-up did not complete 12w follow-up.

Of the 125 consented women, 73 (58.4%) provided data. Data completion rates were similar in both arms, with just over half of participants completing data at 12w and under half at 6m (Table 2). PP analysis showed >70% data completion at 12w, and >60% at 6m.

More participants with secondary and higher education levels completed follow-up forms than those with no formal education (Supplementary Table 2). Attendance was not influenced by education level.

Twenty women and eight CFs were interviewed. Literacy was highlighted as a key barrier to achieve data completion, as participants needed assistance to fill the required forms. While most participants did not report the amount of paperwork related to being part of a trial as burdensome, this was remarked at interview by CFs (see Supplementary Table 3).

### Participant Outcomes

More participants were abstinent from smoking (corroborated by saliva tests) in the intervention group at 12w, which persisted at 6m (Table 3). Similar results were obtained with self-report data only at 12w. The percentage of participants who were abstinent at both timepoints was similar in both arms. Results were replicated in the PP analysis. In the intervention group, 12/15 (80%) abstinent women at 12w reported being smokers for over 25 years compared to 4/8 (50%) in the control arm. Furthermore, 11/15 (73%) abstinent women in the intervention arm were GMS cardholders compared to 4/8 (50%) in the control arm.

**Table 3.** Smoking Status After Treatment

			Intervention	Control	Total
Measure		Smoking status	*n* (%)	*n* (%)	*n* (%)
ITT *n* = 125	12w	Abstinence^a^	15 (23.1)	8 (13.3)	23 (18.4)
		Continued smoking	50 (76.9)	52 (86.7)	102 (81.6)
	6m	Abstinence^a^	9 (13.8)	7 (10.8)	16 (12.8)
		Continued smoking	56 (86.2)	53 (89.2)	109 (87.2)
	Abstinent^a^ at both 12w and 6m		7 (10.8)	6 (10.0)	13 (10.4)
	12w	Self-reported abstinence	17 (26.1)	10 (16.7)	27 (21.6)
		Continued smoking	48 (73.9)	50 (83.3)	98 (78.4)
	6m	Self-reported abstinence	10 (15.4)	10 (16.7)	20 (16)
		Continued smoking	55 (84.6)	50 (83.3)	105 (84)
	Self-reported abstinence at both 12w and 6m		8 (12.3)	7 (11.7)	15 (12)
PP *n* = 89	12w	Abstinence^a^	15 (30)	8 (20)	23 (25.8)
		Continued smoking	35 (70)	31 (80)	66 (74.2)
	6m	Abstinence^a^	9 (18)	7 (18)	16 (18)
		Continued smoking	41 (82)	32 (82)	73 (82)
	Abstinent ^a^ at both 12w and 6m		7 (14)	6 (15.4)	13 (14.6)
	12w	Self-reported abstinence	17 (34)	10 (25.6)	27 (30.3)
		Continued smoking	33 (66)	29 (74.4)	62 (69.6)
	6m	Self-reported abstinence	10 (20)	10 (25.6)	20 (22.5)
		Continued smoking	40 (80)	29 (74.4)	69 (77.5)
	Self-reported abstinence at both 12w and 6m		8 (16)	7 (17.9)	15 (16.8)
		**Intervention**		**Control**	
Mean % change in no. daily cigarettes^b^		Mean (±SD; 95% CI)		Mean (±SD; 95% CI)	
ITT *n* = 125	From Baseline to 12w	−42.2 (47.7; −54.0 to −30.4)		−28.8 (40.9; −39.4 to −18.2)	
	From Baseline to 6m	−45.0 (45.6; −56.3 to −33.7)		−28.1 (40.0; −38.4 to −17.7)	
PP *n* = 89	From Baseline to 12w	−54.9 (47.5; −68.4 to −41.4)		−44.3 (43.5; −58.4 to −30.2)	
	From Baseline to 6m	−58.5 (43.7; −70.9 to −46.1)		-43.2 (42.6; −57 to −29.4)	
		**Intervention**		**Control**	
Attendance as per smoking status		Mean (±SD)	Median (IQR)	Mean (±SD)	Median (IQR)
12w	Abstinence^a^	9.7 (1.5)	10 (8–11)	5.5 (1.6)	5.5 (5–6)
	Continued smoking	3.8 (3.6)	4 (0–7)	2.1 (2.2)	2 (0–4)
6m	Abstinence^a^	10.2 (1.3)	10 (10–11)	5.9 (1.3)	6 (5.5–6.5)
	Continued smoking	4.4 (3.8)	4 (0–8)	2.1 (2.2)	2 (0–4)
Abstinent^a^ at both 12w and 6m		10.4 (1.4)	10 (10–11.5)	5.8 (1.5)	5.5 (5–7)

ITT = intention-to-treat analysis, including *n* = 65 in intervention and *n* = 60 in control arms; PP = per-protocol analysis, including *n* = 50 in intervention and *n* = 39 in control arms.

^a^Abstinence corroborated by saliva tests.

^b^Mean % change in number of daily cigarettes was based on original and imputed data.

The mean percentage reduction in daily cigarettes smoked between baseline and 12w and baseline and 6m was higher in the intervention arm (Table 3). Women in each trial arm who continued to smoke at 12w attended fewer sessions that those who became abstinent. Most intervention participants took NRT during treatment (Supplementary Table).

Twenty-four participants reported the procedure to collect a saliva sample as comfortable or very comfortable at 12w [I: 17/25 (68%); C: 7/15 (47%)], and 11 reported it as uncomfortable or very uncomfortable [I: 8/25 (32%); C: 3/15 (20%)]. Eight women who self-reported abstinence at either endpoint were unable to provide sufficient saliva; two commented that the swab to collect saliva was very large, and two considered the procedure as too unpleasant. At interview most participants described the procedure as acceptable, although challenges were noted (Supplementary Table 3).

Thirty-eight percent of participants reported at 12w that the voucher was an important/ very important incentive to data completion (Supplementary Table 5).

Women in both arms reported improved physical and mental health at follow-ups (Supplementary Table 6).

### Acceptability, Appropriateness, and Feasibility and Fidelity of WCQ Intervention Delivery

The average acceptability, appropriateness, and feasibility scores for WCQ intervention delivery were 4.25/5 or higher, (Supplementary Table 7).

Of the 82 planned activities 69, (83.8%) were reported as completed across the 12 WCQ sessions, 87.1%, (sessions 1–6); 71.3% (sessions 7–12) (Supplementary Table 7).

### Progression to DT

Recruitment of four matched district pairs was successfully achieved. The overall target of 194 consented women was not reached. As a consequence, there were insufficient data to estimate the intra-cluster correlation. However, women were recruited in four waves, with each wave iteratively adapting their recruitment strategy and the final wave4 reached the expected recruitment rate. The retention target of 120 women (60 per arm) at follow-up was not achieved.

## Discussion

This research demonstrated that recruitment of SED women to a smoking cessation pilot trial delivered in their local community setting was for the most part feasible, through the combined effort of community, voluntary and statutory stakeholders. Although the overall recruitment target of 194 women was not reached, the target of 50% low-income women was exceeded. Trial-related processes were acceptable to CFs, who also delivered the intervention with high fidelity. Evidence on the acceptability of trial processes by participants was mixed. Although satisfactory engagement with stop-smoking treatments was achieved, retention at 12w was much less than the expected 60 participants per arm, which may indicate a lack of acceptability of the trial or intervention. Likely reasons for this are discussed in full in the complementary process evaluation. Low literacy was identified as a root cause of this attrition as those with secondary or higher education had higher data completion rates at follow-up. However, women with all education levels had similar attendance rates, suggesting overall intervention acceptability. The pilot trial was not powered to detect differences in abstinence rates between groups, however, some indication of effect in favor of the intervention group at 12w was observed.

Eight previous definitive smoking cessation RCTs in low-income populations have exclusively recruited women.^17–23,34^ These trials differ from this RCT in that most recruited women from healthcare settings located in SED areas.^18–21,34^ Two trials by Solomon et al.,^22,23^ recruited from the general population using flyers resulting in an eligibility rate of just 50% of registered women. A previous RCT of low-income women, (Andrews et al.,)^17^ the only other tailored smoking cessation RCT to include group support, also used a Community-based Participatory Research approach,^35^ which positively impacted on recruitment of African-American women smokers. Their group treatment^17^ was shorter (6 weeks) than WCQ, it was delivered by a smoking cessation specialist,^17,35^ and was followed by individual home visits by a lay community health worker.^17^ The few other trials that involved trained lay advisers, delivered shorter individual interventions.^22,23,34^

In our study, the accessible community locations for intervention delivery, the high determination to quit reported at baseline, and support from family or friends to help quit smoking, may have facilitated engagement.^36,37^ Average attendance in each arm was <50% of sessions delivered, but surpassed 70% among those who engaged, similar to the 4/6 sessions reported by Andrews et al., who also reported higher abstinence rates with attendance at group sessions.^17^

Group interventions have had a long history of low attendance.^38,39^ Achieving high participation in group settings is known to be challenging, and it may depend on motivation to quit, and on the availability of time and effort in attending the meetings.^38^ Attendance to an in-person stop-smoking program, particularly in a group context may have elicited feelings of shame and guilt among participants. These may be linked to the pressure to succeed and to admitting being a smoker with failed previous quit attempts, which constitute important barriers to receiving support.^40^

These barriers related to delivery of an in-person program may also have influenced retention at follow-up, which was slightly higher in the control arm. The previous RCTs (above) showed higher retention rates, 80%–94% at 3 months^19,22^ and 75%–92% at 6 months.^20–22^ Important differences with our trial were the clinical setting for initial delivery and structured regular follow-up with participants by phone or at home as a part of intervention delivery.^18–21,34^

Our particular population has not been studied in this way before and therefore the attrition is most likely related to smoking status which is the norm for equivalent trials and might be as expected for these communities which have endured decades of deprivation. More than half of our participants lived with another smoker, another well-recognized factor in attrition from cessation aids.^41^

Mixed-methods data from our trial highlighted low literacy as a key barrier to retention at follow-up. Less literate persons may refuse to participate in research activities that might challenge or expose their literacy skills,^42^ which may have in part accounted for early dropouts and low retention rates. Low literacy is often accompanied by feelings of shame and reluctance to disclose reading difficulties, which may act as barriers to seeking help.^43^ Although all forms were adapted for low literacy levels and women received assistance in data completion, additional GDPR introduced during the data collection period demanded more from women in terms of literacy. Participants and CFs highlighted at interview the need to avoid any self-administered material.^44^

In addition to low literacy, the voucher used for participant compensation may have been too low to encourage sustained participation. Previous smoking cessation trials recruiting SED women showing high retention used higher financial incentives than in our study which increased as the study progressed.^17,19,20,23,34^

Access to NRT without charge undoubtedly contributed to higher abstinence rates for WCQ participants. Notably, abstinence rates were higher in GMS women. The lack of free access to NRT in smoking cessation programes is recognized by the World Health Organization Framework Convention on Tobacco Control and needs to be overcome in many countries, including Ireland.^5^

Evidence is limited for the most effective implementation strategies^45^ to encourage sustained participation in RCTs.^46^ Our retention strategies (repeated contact for appointments, data collection at convenient locations, and financial incentive at follow-ups) were insufficient to prevent attrition. Hence, the contextual findings from other studies may not be directly transferable to the general population of white Irish SED women smokers. Future analyses of qualitative data on the acceptability of the WCQ intervention by participants will allow a deeper understanding of the factors linking context, mechanism, and outcome.

### Strengths and Limitations

Key strengths of our study were the use of a Community-based Participatory Research approach, a geo-cluster design, remote randomization, a comparison group receiving an enhanced smoking cessation treatment, the use of mixed methods in evaluation of trial feasibility, and the conduct of a high-quality methodological study including blinding of those enrolling participants and of trial statisticians. From the viewpoint of CFs, delivery of WCQ was highly acceptable, appropriate, and feasible using validated scales.^33^ High fidelity to intervention delivery was achieved. The legacy of a community-based structure will facilitate future scale-up and integration of the program into the HSE once (cost)-effectiveness is determined in a future DT.

High attrition is the biggest challenge in the design of a DT. An important methodological limitation is that we were unable to interview women who dropped out of the study. Multiple efforts were made to contact women and collect reasons to dropout at each point (between registration and randomization, between sessions, and at follow-ups). Most participants could not be contacted. We acknowledge that we are likely to have interviewed a biased sample of more literate women and that the high dropout rate among those not interviewed may indicate a lack of acceptability of the trial or program.

There were differences in the baseline smoking behavior of WCQ participants which would be expected to attenuate any intervention effect. In cluster RCTs with geo-cluster designs, it may be more difficult to achieve balanced characteristics between groups.^47^ The high dropout rates observed prior to randomization may indicate that these women were not sufficiently motivated to participate in the trial.^48^ However, no differences in registration characteristics were noted in those who withdrew from the trial. Recruitment and delivery during holiday periods were important barriers that could not be avoided within the trial resources and timelines.

The geo-cluster design was intended to incur a lower risk of contamination between participants in each group than if women had been individually randomized.^49^ The higher dropout rates in the control arm between randomization and treatment start were in part due to a small number of women stating a preference for the group intervention.

Other potential source of bias may have been social desirability bias arising from the self-administration of the AIM-IAM-FIM questionnaires and of the fidelity checklists, as the CFs may have overestimated their own performance. The low number of respondents (eight CFs) may have also influenced their responses as they may have perceived that their answers may be potentially identifiable. Social desirability bias may have also resulted from the CFs own beliefs about the potential benefits of WCQ.^50^ Alternative methods to assess fidelity such as direct observation or session recording may be useful to address this limitation.

As this was a pilot trial, it was not designed to determine effectiveness but to demonstrate whether the direction of intervention effect was in the expected (positive) direction. The control condition recruiting women to the HSE standard tobacco cessation program was enhanced for the pilot trial to be structurally equivalent to WCQ.^28^ Hence, it was designed to demonstrate evidence in expected direction under the toughest possible conditions. However, we acknowledge that effectiveness has not been fully tested in this pilot trial and that more evidence is needed to support any further conclusion.

Our data indicate the need to modify certain intervention components to guarantee the conduct of a future DT. An extended community engagement period to optimize recruitment will be required with registration of up to twice as many participants to achieve the target for consented women. Additional implementation strategies to improve retention will be needed. These include greater support for women with low literacy to complete data at follow-ups, additional training for CFs in strategies to address low literacy, greater simplification of all trial-related forms, a comprehensive participant tracking strategy with structured SMS messaging,^44^ and potentially higher and incremental participant compensation. Direct observation of saliva sampling will also be needed.

## Supplementary Material

A Contributorship Form detailing each author’s specific involvement with this content, as well as any supplementary data, are available online at https://academic.oup.com/ntr.

ntab242_suppl_Supplementary_Table_1

ntab242_suppl_Supplementary_Table_2

ntab242_suppl_Supplementary_Table_3

ntab242_suppl_Supplementary_Table_4

ntab242_suppl_Supplementary_Table_5

ntab242_suppl_Supplementary_Table_6

ntab242_suppl_Supplementary_Table_7

ntab242_suppl_Supplementary_Materials_S1

ntab242_suppl_Supplementary_Materials_S2

ntab242_suppl_Supplementary_Materials_S3

ntab242_suppl_Supplementary_Taxonomy_Form

## Data Availability

Anonymized individual participant quantitative data collected during the trial will be available after publication from the corresponding author.
