# Supplementary material for: Peer-Delivery of a Gender-Specific Smoking Cessation Intervention for Women Living in Disadvantaged Communities in Ireland We Can Quit2 (WCQ2)—A Pilot Cluster Randomized Controlled Trial
Source: Nicotine Tob Res. 2021 Nov 20;24(4):564–73. doi: 10.1093/ntr/ntab242 (PMC8887585; doi:10.1093/ntr/ntab242)
Supplement: ntab242_suppl_Supplementary_Table_1 [file ntab242_suppl_supplementary_table_1.docx]

**Supplementary Table 1. Baseline characteristics of all randomised participants.**

| Socio-demographics | Intervention  (n=65) | Control  (n=60) | Total  (n=125) |
| --- | --- | --- | --- |
| Age |  |  |  |
| Mean (SD) | 50.0 (±11.6) | 45.8 (±12.1) |  |
| Marital status |  |  |  |
| Married / Cohabiting / civil partnership | 34 (52.3) | 23 (38.3) | 57 (46) |
| Single (never married) | 11 (17) | 23 (39) | 34 (27) |
| Other (separated, divorced, widowed) | 20 (30.7) | 13 (21.6) | 33 (27) |
| Children at home (0-18 years) | | | |
| No children at home | 38 (59) | 39 (65) | 77 (62) |
| 1 or more | 27 (41) | 21 (35) | 48 (38) |
| Education | | | |
| No formal / Primary / Lower | 31 (48) | 21 (35) | 52 (42) |
| Secondary / Technical or Vocational / Completed Apprenticeship | 20 (31) | 23 (38) | 43 (34) |
| Degree (Diploma, Masters, PhD) | 14 (22) | 16 (27) | 30 (24) |
| Employment | | | |
| Full-time | 20 (31) | 18 (31) | 38 (31) |
| Part time | 15 (23) | 14 (24) | 29 (23) |
| Not in paid employment | 30 (46) | 27 (46) | 57 (45) |
| Holds a medical or GP card | | | |
| Yes | 41 (63) | 35 (60) | 76 (62) |
| No | 24 (37) | 23 (40) | 47 (38) |
| Smoking behaviour | | | |
| Daily cigarettes |  |  |  |
| Mean (SD) | 18.0 (±7.0) | 19.0 (±9.9) |  |
| Length of time smoking |  |  |  |
| More than 25 years | 50 (78) | 36 (61) | 86 (70) |
| Between 5-15 / 15-25 years | 14 (21.5) | 23 (38.3) | 37 (30) |
| Reasons for smoking | | | |
| Habit / Addicted | 35 (53.8) | 26 (43.3) | 61 (48.8) |
| For pleasure / to cope / both | 22 (34) | 29 (48.3) | 51 (40.8) |
| Other | 8 (12) | 5 (8.3) | 13 (10.4) |
| Time after waking before first cigarette | | | |
| Within 5 minutes | 40 (62) | 30 (50) | 70 (56) |
| 6-30 minutes | 12 (18) | 16 (27) | 28 (22) |
| After 30 minutes | 13 (20) | 14 (23) | 27 (22) |
| Determination to give up smoking | | | |
| Not at all / Quite determined | 23 (35) | 18 (30) | 41 (33) |
| Very or extremely determined | 42 (65) | 42 (70) | 84 (67) |
| Using NRT/bupropion/varenicline/e-cigarettes at baseline | | | |
| Yes | 14 (22) | 9 (16) | 23 (19) |
| No | 49 (78) | 49 (84) | 98 (81) |
| Support to help stop smoking | | | |
| Yes | 58 (89) | 50 (83) | 108 (86) |
| No | 7 (11) | 10 (17) | 17 (14) |
| Presence of regular smokers at home | | | |
| Yes | 34 (52) | 31 (52) | 65 (52) |
| No | 31 (48) | 29 (48) | 60 (48) |
| Cohabitants who smoke regularly* | |  |  |
| Spouse | 22 (63) | 13 (37) | 35 (100) |
| Child / Other | 14 (40) | 21(60) | 35 (100) |

Data are n (%) unless specified.

*Participants could select more than one option. Percentages represents the total answers for each option. The total row represents the total number of answers in each category.
