# Supplementary material for: Peer-Delivery of a Gender-Specific Smoking Cessation Intervention for Women Living in Disadvantaged Communities in Ireland We Can Quit2 (WCQ2)—A Pilot Cluster Randomized Controlled Trial
Source: Nicotine Tob Res. 2021 Nov 20;24(4):564–73. doi: 10.1093/ntr/ntab242 (PMC8887585; doi:10.1093/ntr/ntab242)
Supplement: ntab242_suppl_Supplementary_Table_2 [file ntab242_suppl_supplementary_table_2.docx]

**Supplementary table 2. GMS status, attendance and data completion rates of participants as a function of the education level reported at baseline.**

|  | | **Education** | | | |
| --- | --- | --- | --- | --- | --- |
|  |  | **No formal/ Primary/ Lower** | | **Secondary or higher** | |
|  |  | **Intervention** | **Control** | **Intervention** | **Control** |
| **GMS status** | n (%) | 18/37 (48.6) | 19/37 (51.3) | 23/39 (59) | 16/39 (41) |
| **Attendance** | Mean (SD) | 4.8 (4.6) | 2.4 (2.5) | 5.5 (3.5) | 2.63 (2.32) |
|  | Median (IQR) | 3 (0-10) | 2 (0-5) | 5.5 (3-8) | 2 (0-4) |
| **Data completion** | n (%) | 11/31 (35.5) | 7/21 (33.3) | 17/34 (50) | 18/39 (46.1) |
