# Supplementary material for: Peer-Delivery of a Gender-Specific Smoking Cessation Intervention for Women Living in Disadvantaged Communities in Ireland We Can Quit2 (WCQ2)—A Pilot Cluster Randomized Controlled Trial
Source: Nicotine Tob Res. 2021 Nov 20;24(4):564–73. doi: 10.1093/ntr/ntab242 (PMC8887585; doi:10.1093/ntr/ntab242)
Supplement: ntab242_suppl_Supplementary_Table_3 [file ntab242_suppl_supplementary_table_3.docx]

**Supplementary Table 3.** **Participants and community facilitators quotes obtained at interview.**

| **Theme** | **Quote** |
| --- | --- |
| Literacy | *I don’t write very well. So, what we were doing was em (CF) would help us with the filling out so you don’t feel embarrassed because it is embarrassing when they’re asking us to fill in stuff which I can’t do and (CF) would say she’ll do it later for us if we wanted to or what we could do was bring it home and [partner] would fill it in for me. I started off though by saying that I had forgot me glasses.* (Interview P0013) |
| Volume of paperwork | *CF: I know it has to start somewhere but given it was the research programme and everything, there was lots of extra stuff to do and people were being very picky, and you know that kind of thing. There was a lot of extra paperwork than I wouldn’t have done before.* (Interview CF) |
| Saliva collection | *It took me ages to get a bit (of saliva)! It was a bit uncomfortable, but it was grand*. (Interview P0026) |

Abbreviations: P, participant; CF, community facilitator.
