# Supplementary material for: Peer-Delivery of a Gender-Specific Smoking Cessation Intervention for Women Living in Disadvantaged Communities in Ireland We Can Quit2 (WCQ2)—A Pilot Cluster Randomized Controlled Trial
Source: Nicotine Tob Res. 2021 Nov 20;24(4):564–73. doi: 10.1093/ntr/ntab242 (PMC8887585; doi:10.1093/ntr/ntab242)
Supplement: ntab242_suppl_Supplementary_Table_4 [file ntab242_suppl_supplementary_table_4.docx]

**Supplementary Table 4. Nicotine replacement therapy (NRT) use during treatment.**

| NRT use | Intervention  (n=65) | Control  (n=60) | Total  (n=125) |
| --- | --- | --- | --- |
| Mean weeks medication recommended (±SD) | 7.0 (3.7) | 6.9 (4.0) |  |
| Took NRT | 32 (49.2) | 17 (28.3) | 49 (39) |
| Used NRT for the full recommended time | 20/32 (62.5) | 11/17 (64.7) | 31/49 (63.2) |
| Kept to recommended dose | 21/32 (65.6) | 10/17 (58.8) | 31/49 (63.2) |
| Types used* |  |  |  |
| Patches | 32 | 17 | 49 |
| Inhalator | 7 | 9 | 16 |
| Gum | 9 | 3 | 12 |
| Lozenge | 6 | 5 | 11 |

Data are n (%) unless specified. *Participants may select more than one type of NRT.
