# Supplementary material for: Peer-Delivery of a Gender-Specific Smoking Cessation Intervention for Women Living in Disadvantaged Communities in Ireland We Can Quit2 (WCQ2)—A Pilot Cluster Randomized Controlled Trial
Source: Nicotine Tob Res. 2021 Nov 20;24(4):564–73. doi: 10.1093/ntr/ntab242 (PMC8887585; doi:10.1093/ntr/ntab242)
Supplement: ntab242_suppl_Supplementary_Table_5 [file ntab242_suppl_supplementary_table_5.docx]

| **Supplementary table 5. Importance of voucher as incentive to continue in the trial as reported at 12w follow-up.** | **Intervention** | **Control** | **Total** |
| --- | --- | --- | --- |
| **Importance of voucher** | n (%) | n (%) | n (%) |
| Very important / Important | 13/30 (43.3) | 9/28 (32.1) | 22 (37.9) |
| Neither important nor unimportant | 4/30 (13.3) | 7/28 (25.0) | 11 (19.0) |
| Unimportant / Very unimportant | 13/30 (43.3) | 11/28 (39.3) | 24 (41.4) |
| Total who provided data | 30/65 (46.1) | 28/60 (46.6) | 58 (46.4) |
