# Supplementary material for: Peer-Delivery of a Gender-Specific Smoking Cessation Intervention for Women Living in Disadvantaged Communities in Ireland We Can Quit2 (WCQ2)—A Pilot Cluster Randomized Controlled Trial
Source: Nicotine Tob Res. 2021 Nov 20;24(4):564–73. doi: 10.1093/ntr/ntab242 (PMC8887585; doi:10.1093/ntr/ntab242)
Supplement: ntab242_suppl_Supplementary_Table_6 [file ntab242_suppl_supplementary_table_6.docx]

**Supplementary table 6. Percentage improvement in physical and mental health status as measured by SF-12 instrument.**

|  | | | Intervention | | Control | |
| --- | --- | --- | --- | --- | --- | --- |
| Health status | | **Period** | n (%) | 95% CI | n (%) | 95% CI |
| ITT n=125 | Improvement in physical health | From baseline to 12w | 23 (35.4) | 28.3-47.5 | 17 (28.3) | 18.5-40.7 |
|  |  | From baseline to 6m | 23 (35.4) | 24.9-47.5 | 22 (36.6) | 25.6-49.3 |
|  | Improvement in mental health | From baseline to 12w | 14 (21.5) | 13.3-32.9 | 17 (28.3) | 18.5-40.7 |
|  |  | From baseline to 6m | 20 (30.7) | 20.8-42.7 | 15 (25) | 15.8-37.2 |
| PP n=89 | Improvement in physical health | From baseline to 12w | 23 (46) | 33-59.6 | 17 (43.6) | 29-59 |
|  |  | From baseline to 6m | 23 (46) | 33-59.6 | 22 (56.4) | 41-70 |
|  | Improvement in mental health | From baseline to 12w | 14 (28) | 17-41.7 | 17 (43.6) | 29-59 |
|  |  | From baseline to 6m | 20 (40) | 28-53.8 | 15 (38.4) | 25-54 |

ITT: Intention to treat analysis, including n=65 in intervention and n=60 in control arms. PP: Per-protocol analysis, including n=50 in intervention and n=39 in control arms.
