# Supplementary material for: Peer-Delivery of a Gender-Specific Smoking Cessation Intervention for Women Living in Disadvantaged Communities in Ireland We Can Quit2 (WCQ2)—A Pilot Cluster Randomized Controlled Trial
Source: Nicotine Tob Res. 2021 Nov 20;24(4):564–73. doi: 10.1093/ntr/ntab242 (PMC8887585; doi:10.1093/ntr/ntab242)
Supplement: ntab242_suppl_Supplementary_Table_7 [file ntab242_suppl_supplementary_table_7.docx]

**Supplementary Table 7.** **Acceptability, appropriateness and feasibility scores*, and activities self-reported as delivered in each session by Community Facilitators.**

|  | Acceptability | Appropriateness | Feasibility | |
| --- | --- | --- | --- | --- |
| Mean (±SD) | 4.46 (0.5) | 4.25 (0.44) | 4.5 (0.51) | |
|  | **Fidelity** | | | |
|  | Activities planned | Activities delivered^#^ | | |
| Session | n | Mean (±SD) | | % |
| 1 | 13 | 11.8 (1.3) | | 91.3 |
| 2 | 10 | 9.5 (1.0) | | 95.0 |
| 3 | 11 | 9 (2.1) | | 81.8 |
| 4 | 11 | 9.8 (1.4) | | 89.8 |
| 5 | 10 | 9.5 (0.5) | | 95.0 |
| 6 | 10 | 6.8 (3.3) | | 68.8 |
| 7 | 4 | 3.2 (0.6) | | 81.3 |
| 8 | 2 | 1.6 (0.5) | | 81.3 |
| 9 | 2 | 1.7 (0.5) | | 87.5 |
| 10 | 2 | 1.7 (0.5) | | 87.5 |
| 11 | 5 | 2.5 (2.3) | | 50.0 |
| 12 | 2 | 1.2 (0.9) | | 62.5 |
| Total | **82** | **68.7 (4.0)** | | **83.8** |

*Seven of eight CFs completed the acceptability, appropriateness and feasibility questionnaires.

^#^Activities from two missed sessions (sessions 6 and 11 in waves 3 and 4, respectively) were analysed as if delivered in the original planned session.
