# Supplementary material for: Peer-Delivery of a Gender-Specific Smoking Cessation Intervention for Women Living in Disadvantaged Communities in Ireland We Can Quit2 (WCQ2)—A Pilot Cluster Randomized Controlled Trial
Source: Nicotine Tob Res. 2021 Nov 20;24(4):564–73. doi: 10.1093/ntr/ntab242 (PMC8887585; doi:10.1093/ntr/ntab242)
Supplement: ntab242_suppl_Supplementary_Materials_S1 [file ntab242_suppl_supplementary_materials_s1.pdf]

WCQ2 Community Facilitators

Checklist and Diary [1]

Dear Community Facilitator,

Here is the Checklist and Diary, which related to the WCQ2 programme, that we would like you to complete on a weekly basis. If you are co-facilitating sessions then please complete this Checklist and Diary together.

Checklist

We would like to see which topics were covered in the sessions each week. Please confirm which parts of the session that you were able to cover by placing a tick beside them within the Checklist. If you did not cover a part of the session then please leave it blank.

Diary

We would like your thoughts about how each session went. Please take a moment to reflect on each session by completing the questions in the Diary section.

Collection

We will collect this information after week 6 and after week 12. You will get a copy to keep to help you with the planning of the remaining sessions.

Thank you very much.

Your WCQ2 Team.

[1] This is a modified version of the original form excluding diaries related questions.

Session 1 – Preparing to Quit<br>Date of Delivery:	
Please tick all activities/worksheets covered:	√
Welcome and introduction	
WCQ programme information	
Hopes, fears and expectations	
Ground rules	
Brainstorm: reasons for quitting	
Overview of one to one sessions	
Personal Carbon Monoxide chart	
Smoking habit quiz	
Readiness to quit smoking	
Smoking diary	
Weekly progress chart	
Participants packs distributed	
Home exercise	

Session 2 – Preparing to Quit<br>Date of Delivery:	
Please tick all activities/worksheets covered:	√
The Decisional Balance exercise and discussion	
Carbon Monoxide, smoking and your health	
Setting quit date	
Brainstorm: what helps to quit	
Evidence-based cessation methods	
Nicotine and NRT	
Fagerstrom tolerance questionnaire	
Top 10 tips for successful quitting	
Home exercise: what influences my health?	
Evaluation	

Session 3 – Benefits of Quitting<br>Date of Delivery:	
Please tick all activities/worksheets covered:	√
Feedback on week: what influences my health	
Health benefits of quitting	
Non-evidence based methods	
Is smoking an addiction?	
e-cigarettes	
The benefits of quitting factsheet	
Breaking the links with cigarettes	
Going smoke free - the 4 Ds	
Relaxation	
Home exercise: My quitting plan Worksheet, Record of nicotine cravings Worksheet, Tips for avoiding relapse	
Evaluation	

**Session 4 – Counting the cost of smoking**<br>**Date of Delivery:**	
Please tick all activities/worksheets covered:	√
Review of week	
Review personal coping plan	
Counting the cost of smoking	
Brainstorm: withdrawal symptoms	
Brainstorm: what is stress?	
Understanding stress factsheet	
Managing stress: what can we do?	
Relaxation	
One to one	
Home exercise: overcoming challenges	
Evaluation	

Session 5 – Healthy eating when quitting<br>Date of Delivery:	
Please tick all activities/worksheets covered:	√
Feedback on week	
Discussion: overcoming challenges worksheet	
Discussion: agree topics for discussion in 2nd half of programme	
Quitting smoking doesn't have to be a weighty matter!	
Food pyramid	
Healthy eating when quitting Worksheet	
Quit smoking and eat healthily Factsheet	
Relaxation exercise	
One to one	
Evaluation	

Session 6 – Planning for the future<br>Date of Delivery:	
Please tick all activities/worksheets covered:	√
Feedback on week	
Happy face – Sad face exercise	
Discussion on relapse and successful quitting	
Discussion on plan for next 6 weeks	
Brainstorming: supporting journey from smoker to non-smoker	
Success and relapse prevention	
Managing relapse: case studies	
One to one	
Home exercise: how to be happy! Top tips	
Evaluation	

Session 7 – Celebrate Achievements & Health checks<br>Date of Delivery:	
Please tick all activities/worksheets covered:	√
The art of celebrating: reflections of your quitting journey	
Worksheet/template 1 for celebrating achievements/health check	
Worksheet 3 Calculate your mileage	
Evaluation & close	

**Session 8 – Celebrate Achievements & Health Checks**<br>**Date of Delivery:**	
Please tick all activities/worksheets covered:	√
Worksheet women's health and identity	
Optional activities (*please list*):	

Session 9 – Staying quit<br>Date of Delivery:	
Please tick all activities/worksheets covered:	√
Staying quit Worksheet alcohol diary	
Optional activities (*please list*):	

Session 10 – Staying quit<br>Date of Delivery:	
Please tick all activities/worksheets covered:	√
Staying quit Worksheet: What's in tobacco smoke wordsearch	
Optional activities (*please list*):	

**Session 11 – We Can Quit review<br>Date of Delivery:**	
Please tick all activities/worksheets covered:	√
Factsheet: be active	
Worksheet: barriers and solutions	
Worksheet: my tips for getting active	
Worksheet: my plan to be more active	
Optional activities (*please list*):	

**Session 12 – Closing session<br>Date of Delivery:**	
Please tick all activities/worksheets covered:	√
Presentation of certificates	
Optional activities (*please list*):	
