# Supplementary material for: Peer-Delivery of a Gender-Specific Smoking Cessation Intervention for Women Living in Disadvantaged Communities in Ireland We Can Quit2 (WCQ2)—A Pilot Cluster Randomized Controlled Trial
Source: Nicotine Tob Res. 2021 Nov 20;24(4):564–73. doi: 10.1093/ntr/ntab242 (PMC8887585; doi:10.1093/ntr/ntab242)
Supplement: ntab242_suppl_Supplementary_Materials_S2 [file ntab242_suppl_supplementary_materials_s2.pdf]

Date:

Participant ID:<br>Site ID:

We Can Quit2 Baseline Data Monitoring Form

About this Form

We need to gather background information about each woman on the programme. Your information will be treated with the strictest of confidence. In keeping with national and Trinity College guidelines, we will keep your data in a safe and secure place for 7 years. Only the research team will have access to your data. Your name and contact details will not be used for the study. They will be stored separately and will only be used when a member of the research team contacts you.

Participant information and consent form

Before completing this form, please confirm that you have received, read and understood the **participant information leaflet**?

Yes ☐ No ☐ *(if no, make sure to receive and read the leaflet before proceeding)*

Before completing this form, please confirm that you have completed the **consent form?**

Yes ☐ No ☐ *(if no, make sure to complete the consent form before proceeding)*

Have you heard of the We Can Quit programme?

Yes ☐ No ☐

SECTION 1: ABOUT YOU

In this section, we would like to ask some questions about you, your living arrangements and smoking behaviour.

**First name:**	**Surname:**

1. Are you in paid employment? ☐ Yes full-time ☐ Yes part-time ☐ Carer ☐ No not in paid employment

If 'yes', what is your occupation? ……………………………………………………

If 'no', which of the categories below best describes you?

☐ Unemployed ☐ Full-time student or pupil ☐ Sick/disabled and unable to work
☐ Retired ☐ Homemaker/full time parent/carer ☐ Other (*write in*)……………………………….

2. How would you describe your background (*tick one only*):

White
☐ Irish
☐ Irish Traveller
☐ Any other White background

Black or Black Irish
☐ African
☐ Other Black background

Asian or Asian Irish
☐ Chinese
☐ Any other Asian background

Other, including mixed background (*write in*)
……………………………..
☐ Prefer not to say

3. **Which best describes your education/training?**<br><br>☐ No formal education/Primary education/Lower Secondary (Junior cert.)<br><br>☐ Upper Secondary (Leaving cert.)/ Technical or Vocational/Completed Apprenticeship<br><br>☐ Degree (Postgraduate Diploma, Masters, PhD)	4. **How many people (including yourself) live in your home?**<br><br>Adults<br>(aged 18 or over, include yourself)<br><br>………………………………………………………<br><br>Children (aged 15-18)<br><br>………………………………………………………<br><br>Children (aged 0-14)<br><br>………………………………………………………	5. **What is your marital status?**<br><br>☐ Single (never married)<br><br>☐ Married<br>☐ In a registered civil partnership<br>☐ Separated<br>☐ Divorced<br>☐ Widowed<br>☐ Cohabiting
6. **Do you have a GP?** ☐ Yes ☐ No<br>**GP Address** (if willing to provide)<br><br>……………………………………………….<br><br>……………………………………………….	7. **Do you currently have a full medical card?**<br>☐ Yes ☐ No<br>8. **Do you currently have a GP visit only card?**<br>☐ Yes ☐ No	9. **Would you like us to let your GP know you are taking part in this research study?**<br><br>☐ Yes ☐ No

SECTION 2: SMOKING

This section asks about your smoking.

| 10. **Are you currently using any of the following to try to help you stop smoking?**<br>*(Please tick all that apply)*<br><br>☐ E-cigarette<br>☐ Nicotine replacement (e.g. patch, gums etc.) bought over the counter<br>☐ Nicotine replacement prescribed by GP<br>☐ Champix (varenicline)<br>☐ Zyban (bupropion)<br>☐ Attending a HSE stop smoking support group<br>☐ Attending another support group<br>☐ HSE online support – Quit.ie<br>☐ HSE Quitline – 1800 201 203<br>☐ Attending a stop smoking clinic (HSE)<br>☐ No, I am not using any help<br>☐ Other (describe)…………………. | 10. **Why do you smoke**?<br><br>☐ Mainly for pleasure<br>☐ For pleasure and to cope<br>☐ Mainly to cope<br>☐ Other (write in)<br><br>……………………………………………….<br><br>11. **How long have you been smoking?**<br><br>☐ More than 25 years<br>☐ Between 15-25 years<br>☐ Between 10 and 15 years<br>☐ Between 5 and 10 years<br>☐ Less than 5 years | 12. **On average, how many cigarettes do you usually smoke per day?**<br><br>☐☐ cigarettes per day<br><br>*To be filled in by researcher:*<br>☐ 10 or less<br>☐ 11-20<br>☐ 21-30<br>☐ 31 or more |

**13. How soon after you wake up do you smoke your first cigarette?**<br><br>☐ Within 5 minutes<br>☐ 6-30 minutes<br>☐ 31-60 minutes<br>☐ after 60 minutes	**14. Do you smoke indoors?**<br><br>☐ Yes ☐ No	**15. How determined are you to give up smoking at this attempt?**<br><br>☐ Not at all determined<br>☐ Quite determined<br>☐ Very determined<br>☐ Extremely determined

16. Does anyone that you live with smoke regularly?

☐ Yes
☐ No
☐ Does not apply to me

**If Yes – who?** (*please tick all that apply*)	**Do they smoke indoors?** Yes	No
☐ Spouse/partner	☐	☐
☐ Child	☐	☐
☐ Parent	☐	☐
☐ Sister/brother	☐	☐
☐ Flatmate	☐	☐
☐ Other (*write in*)	☐	☐

17. Do you have anyone who will support you to stop smoking?

☐ Yes ☐ No

If yes - who?
(*please tick all that apply*)
☐ Spouse/partner
☐ Family member
☐ Friend
☐ Work colleagues
☐ Other (*write in*)

………………………………………………..

SECTION 3: YOUR GENERAL HEALTH AND WELLBEING

Just a few questions about your general health and wellbeing.

The following questions ask for your views about your health, how you feel and how well you are able to do your usual activities.

If you are unsure about how to answer any questions, please give the best answer you can and make any of your own comments if you like. Do not spend too much time in answering as your immediate response is likely to be the most accurate.

18. In general, would you say your health is *(Please tick one box)*

Excellent	Very good	Good	Fair	Poor
☐	☐	☐	☐	☐

HEALTH AND DAILY ACTIVITIES

The following questions are about activities you might do during a typical day. Does your health limit you in these activities? If so, how much? *(please tick one box)*

	Yes, limited a lot	Yes, limited a little	No, not limited at all
**19. Moderate activities, such as moving a table, pushing a vacuum cleaner, bowling, or playing golf**	☐	☐	☐
**20. Climbing several flights of stairs**	☐	☐	☐

During the past 4 weeks, have you had any of the following problems with your work or other regular activities as a result of your physical health *(Please answer Yes or No to each question)*		
	Yes	No
**21. Accomplished less than you would like**	☐	☐
**22. Were limited in the kind of work or other activities**	☐	☐

During the past 4 weeks, have you had any of the following problems with your work or other regular activities as a result of any emotional problems (such as feeling depressed or anxious)? *(Please answer Yes or No to each question)*		
	Yes	No
**23. Accomplished less than you would like**	☐	☐
**24. Didn't do work or other activities as carefully as usual**	☐	☐

25. During the past 4 weeks, how much did pain interfere with your normal work (including work both outside the home and housework)? ***(Please tick one box)***

Not at all	A little bit	Moderately	Quite a bit	Extremely
☐	☐	☐	☐	☐

These questions are about how you feel and how things have been with you during the past month. For each question, please give the one answer that comes closest to the way you have been feeling. *(Please tick one box on each line)*

**How much of the time during the last month:**	All of the time	Most of the time	A good bit of the time	Some of the time	A little of the time	None of the time
**26. Have you felt calm and peaceful?**	☐	☐	☐	☐	☐	☐
**27. Did you have a lot of energy?**	☐	☐	☐	☐	☐	☐
**28. Have you felt downhearted and low?**	☐	☐	☐	☐	☐	☐
**29. Has your health limited your social activities (like visiting friends or close relatives)?**	☐	☐	☐	☐	☐	☐

Form completed by:
Name: ………………………………………………………………………

Role: ☐ ICS Staff ☐ Researcher ☐ Participant ☐ Other (write in……………………………….)

Date:

Participant ID:
Site ID:

12 Week We Can Quit2 Follow-Up

SECTION 1: ABOUT THE PROGRAMME

**Participant Name:**	
**Actual Quit Date:** (dd/mm/yyyy)	☐☐ ☐☐ ☐☐☐☐
**Comments:**	

**1. Have you stopped smoking?**<br>Yes – and I'm still not smoking ☐ *Go to Question 2*<br>No – I smoke but I have cut down ☐ *Go to Question 3*<br>Yes – but I relapsed *Go to Question 4* ☐<br>No – I still smoke *Go to Question 5* ☐	**2. If you have stopped smoking, how long has it been since your most recent quit date?**<br>☐ **days** ☐ **weeks**<br>*Go to Question 4*
**3. Have you cut down since starting the programme?**<br>Yes No<br>**How many cigarettes a day do you now smoke?**<br>☐ **cigarettes**<br>*Go to Question 5*	**4. If you stopped smoking and relapsed, how long did you stop for?**<br>☐ **days** ☐ **weeks**<br>**What was the main reason for your relapse?**<br>……………………………………………………………………….<br>**What helped you get back on track?**<br>……………………………………………………………………….
**5. Are you using e-cigarettes?**<br>Yes No<br>**If yes, for how long in total have you been using e-cigarettes?**<br>☐ **days** ☐ **weeks**	**6. What helped you most in trying to stop smoking?** *(please tick all that apply)*<br>NRT ☐<br>Group support ☐<br>One-to-one support ☐<br>Other *(please list below)*: ☐<br>___________________________________
**7. Did you receive a NRT prescription from your GP?**<br>Yes No Not applicable	**8. Did you receive NRT directly from pharmacy without prescription?**<br>Yes No Not applicable
**9. How many weeks was NRT prescribed for?**<br>☐ **weeks** Not applicable	**10. Did you keep to the NRT dosage that was prescribed?** (For example, 21 mg or 14 mg patches)

Did you use it for the full length of time that it was prescribed for?

Yes No Not applicable

Yes No Not applicable

Comments:

11. Did you use a single NRT product or combination (2 types or more)?

Single Combination Not applicable

12. Which type of NRT/other medication did you use?

(see list below and write in code):

☐☐☐☐☐☐☐☐

Please use numbers to show if more than one medication is used consecutively (e.g. NRT Patch is used as well as inhalator would be coded as 2 & 4)

None	Code 1
NRT-Patch	Code 2
NRT-Gum	Code 3
NRT – Inhalator	Code 4
NRT – Lozenge	Code 5
NRT – Mist Spray	Code 6
Champix	Code 7
Zyban	Code 8

13. Which type of behavioural support did you receive? *(see list below and write in code)*:

☐☐☐☐☐☐

Group	Code 1
One-to-one face-to-face support	Code 2
Text message	Code 3
Telephone Support	Code 4
Advice on NRT and letter for GP	Code 5
Other	Code 6

14. Did you receive group support?

Yes No

15. If YES , how many group sessions did you attend:

☐☐

16. How long did the group sessions last on average

1. 0-30 minutes ☐
2. 31-60 minutes ☐
3. 61-90 minutes ☐
4. over 90 minutes ☐

17. Did you receive one-to-one support, i.e. individual support?

Yes No

If so, what type of support did you receive?

*(please tick **all** that apply)*

Face-to-face meeting ☐
Telephone support ☐
Email support ☐
Text support ☐
None ☐
Other:

____

18. How many face to face meetings did you attend?

☐☐

19. How long did the face to face sessions last on average?

1. 0-15 minutes ☐
2. 16-30 minutes ☐
3. 31-60 minutes ☐
4. 61-90 minutes ☐
5. Over 90 minutes ☐

---

20. Were you given information to bring home to read/go through? Yes No

	**21.** What was this? (*passport to quit)*<br>Write in……………………………………………………………

**How satisfied were you with the following:**<br>*(please tick one box per question)*	Very satisfied	Satisfied	Neither satisfied nor dissatisfied	Dissatisfied	Very dissatisfied
**22. Registration process**	☐	☐	☐	☐	☐
**23. Consent process**	☐	☐	☐	☐	☐
**24. Support received from facilitators**	☐	☐	☐	☐	☐
**25. Follow-up process**	☐	☐	☐	☐	☐
**26. Length of the programme**	☐	☐	☐	☐	☐

Comments:

27. How comfortable were you with your saliva sample being taken?

Very comfortable | Comfortable | Unsure | Uncomfortable | Very uncomfortable

Comments:

28. Who was your facilitator *(code only to be recorded):*

29. CO reading:

☐ Refused to give sample

30. Saliva sample taken:

Yes | No, still smoking | No, refused to give sample

SECTION 2: YOUR GENERAL HEALTH AND WELLBEING

Just a few questions about your general health and wellbeing.

The following questions ask for your views about your health, how you feel and how well you are able to do your usual activities.

If you are unsure about how to answer any questions, please give the best answer you can and make any of your own comments if you like. Do not spend too much time in answering as your immediate response is likely to be the most accurate.

31. In general, would you say your health is *(Please tick one box)*

Excellent	Very good	Good	Fair	Poor
☐	☐	☐	☐	☐

HEALTH AND DAILY ACTIVITIES

The following questions are about activities you might do during a typical day. Does your health limit you in these activities? If so, how much? *(please tick one box)*

	Yes, limited a lot	Yes, limited a little	No, not limited at all
**32. Moderate activities, such as moving a table, pushing a vacuum cleaner, bowling, or playing golf**	☐	☐	☐
**33. Climbing several flights of stairs**	☐	☐	☐

During the past 4 weeks, have you had any of the following problems with your work or other regular activities as a result of your <u>physical health</u> *(Please answer Yes or No to each question)*

	Yes	No
**34. Accomplished less than you would like**	☐	☐
**35. Were limited in the kind of work or other activities**	☐	☐

During the past 4 weeks, have you had any of the following problems with your work or other regular activities as a result of any <u>emotional problems</u> (such as feeling depressed or anxious)? *(Please answer Yes or No to each question)*

	Yes	No
**36. Accomplished less than you would like**	☐	☐
**37. Didn’t do work or other activities as carefully as usual**	☐	☐

**38. During the past 4 weeks, how much did pain interfere with your normal work (including work both outside the home and housework)?** ***(Please tick one box)***				
Not at all	A little bit	Moderately	Quite a bit	Extremely
☐	☐	☐	☐	☐

These questions are about how you feel and how things have been with you during the past month. For each question, please give the one answer that comes closest to the way you have been feeling. *(Please tick one box on each line)*

**How much of the time during the last month:**	All of the time	Most of the time	A good bit of the time	Some of the time	A little of the time	None of the time
**39. Have you felt calm and peaceful?**	☐	☐	☐	☐	☐	☐
**40. Did you have a lot of energy?**	☐	☐	☐	☐	☐	☐
**41. Have you felt downhearted and low?**	☐	☐	☐	☐	☐	☐
**42. Has your health limited your social activities (like visiting friends or close relatives)?**	☐	☐	☐	☐	☐	☐

Form completed by:

Name: …………………………………………………………………….

Role: ☐ Researcher ☐ ICS staff ☐ Participant ☐ Other (write ………………………………………….)

Date:

Date:	Participant ID:<br>Site ID:

6 Month We Can Quit2 Follow-Up

SECTION 1: ABOUT THE PROGRAMME

Participant Name:

Actual Quit Date:
(dd/mm/yyyy) ☐☐ ☐☐ ☐☐☐☐

1. Have you received any support since the stop smoking programme ended?
Yes No

If so, what type of support did you receive? *(please tick **all** that apply)*

		Number of sessions
Face-to-face meeting	☐	
Telephone support	☐	
Email support	☐	
Text support	☐	
NRT	☐	
None	☐	
Other *(please list below)*:	☐	

____

Comments:

2. Have you stopped smoking?
Yes – and I'm still not smoking ☐
Go to Question 3
No – I smoke but I have cut down ☐
Go to Question 4
Yes – but I relapsed *Go to Question 5* ☐
No – I still smoke *Go to Question 6* ☐

3. If you have stopped smoking, how long has it been since your most recent quit date?

☐ **days** ☐ **weeks**

Go to Question 6

4. Have you cut down since starting the programme?

Yes No

How many cigarettes a day do you now smoke?

☐ **cigarettes**

Go to Question 6

5. If you stopped smoking and relapsed, how long did you stop for?

☐ **days** ☐ **weeks**

What was the main reason for your relapse?

…………………………………………………………………………….

What helped you get back on track?

…………………………………………………………………………….

**6. Are you using e-cigarettes?**<br>Yes No<br><br>**If yes, for how long in total have you been using e-cigarettes?**<br>☐ **days** ☐ **weeks**	**7. What helped you most in trying to stop smoking?**<br>*(please tick **all** that apply)*<br>NRT ☐<br>Group support ☐<br>One-to-one support ☐<br>Other *(please list below)*: ☐<br>____
**8. How important was the incentive, i.e. the voucher, in deciding to take part in the follow-up?**<br>Very important Important Neither important nor unimportant Unimportant Very unimportant<br>**Please explain:**	
**9. Saliva sample taken:**<br>Yes No, still smoking No, refused to give sample	

SECTION 2: YOUR GENERAL HEALTH AND WELLBEING

Just a few questions about your general health and wellbeing.

The following questions ask for your views about your health, how you feel and how well you are able to do your usual activities.

If you are unsure about how to answer any questions, please give the best answer you can and make any of your own comments if you like. Do not spend too much time in answering as your immediate response is likely to be the most accurate.

10. In general, would you say your health is *(Please tick one box)*

Excellent	Very good	Good	Fair	Poor
☐	☐	☐	☐	☐

HEALTH AND DAILY ACTIVITIES

The following questions are about activities you might do during a typical day. Does your health limit you in these activities? If so, how much? *(please tick one box)*

	Yes, limited a lot	Yes, limited a little	No, not limited at all
**11. Moderate activities, such as moving a table, pushing a vacuum cleaner, bowling, or playing golf**	☐	☐	☐
**12. Climbing several flights of stairs**	☐	☐	☐

During the past 4 weeks, have you had any of the following problems with your work or other regular activities as a result of your physical health *(Please answer Yes or No to each question)*		
	Yes	No
**13. Accomplished less than you would like**	☐	☐
**14. Were limited in the kind of work or other activities**	☐	☐

During the past 4 weeks, have you had any of the following problems with your work or other regular activities as a result of any emotional problems (such as feeling depressed or anxious)? *(Please answer Yes or No to each question)*		
	Yes	No
**15. Accomplished less than you would like**	☐	☐
**16. Didn't do work or other activities as carefully as usual**	☐	☐

17. During the past 4 weeks, how much did pain interfere with your normal work (including work both outside the home and housework)? *(Please tick one box)*

Not at all	A little bit	Moderately	Quite a bit	Extremely
☐	☐	☐	☐	☐

These questions are about how you feel and how things have been with you during the past month. For each question, please give the one answer that comes closest to the way you have been feeling. *(Please tick one box on each line)*

**How much of the time during the last month:**	All of the time	Most of the time	A good bit of the time	Some of the time	A little of the time	None of the time
**18. Have you felt calm and peaceful?**	☐	☐	☐	☐	☐	☐
**19. Did you have a lot of energy?**	☐	☐	☐	☐	☐	☐
**20. Have you felt downhearted and low?**	☐	☐	☐	☐	☐	☐
**21. Has your health limited your social activities (like visiting friends or close relatives)?**	☐	☐	☐	☐	☐	☐

Form completed by:

Name: ………………………………………………………………………….

Role: ☐ Researcher ☐ ICS staff ☐ Participant ☐ Other (write ……………………………………….)

Date:
