# Supplementary material for: Peer-Delivery of a Gender-Specific Smoking Cessation Intervention for Women Living in Disadvantaged Communities in Ireland We Can Quit2 (WCQ2)—A Pilot Cluster Randomized Controlled Trial
Source: Nicotine Tob Res. 2021 Nov 20;24(4):564–73. doi: 10.1093/ntr/ntab242 (PMC8887585; doi:10.1093/ntr/ntab242)
Supplement: ntab242_suppl_Supplementary_Materials_S3 [file ntab242_suppl_supplementary_materials_s3.pdf]

WCQ2 - Semi-structured Interview Guide

1- WCQ women participants

Note to researcher - Before focus group begins:
* Explain the nature of the interview to participant (voluntary, free to stop taking part at any time, research questions, why their perceptions/opinions are important, no right/wrong answers, what the data will be used for)
Housekeeping:

- Recording, transcribed word for word
- Phones on silent
- Thank participant for agreeing to take part
- Start recorder

Note: The blue writing is the questions from the 12-Week FU Quantitative Questionnaire.

Qu 6. Questionnaire: What helped you most in trying to stop smoking? Please rank your 3 choices from 1 to 3 with 1 most helpful) NRT, Group Support, One-to-one support,
Qu1: From your questionnaire, you completed with me (last week) I see you marked … as your number one choice, (*NRT or group support* as worked best)
Can you tell me why you think that was most helpful? ….

Qu2: Is there anything else that you feel worked well or enjoyed in the programme?

a. What did you think of the Group Support?
b. What did it mean to you meeting other smokers who want to quit?
c. What did it mean to you to get free NRT?
d. Can you tell me what the one to one support, such as text messages/telephone calls was like?
e. How did it feel having the social support of other women in the group?
f. The group is facilitated by 2 CF's what was your experience of having the group facilitated this way?
g. Do you set a quit date, 'passport to quit' ? Can you tell me more about that? How did it help you quit? (*Did it help with increasing and maintaining motivation to quit/stay quit)*
h. How suitable was the location of the programme? Was it close to your home?

Qu3: Is there anything about the programme you didn't enjoy/ felt didn't work so well for you?

- How did you find being in a group setting? *(Sometimes I felt that 'my voice' was lost in the group).*
- Thinking back, what did you think of the first session?
- What did you think of the programme being 12 weeks?
- ***Also see Relapse Question if participant brings it up here***

Topics not covered in response to above, ask the following questions:
Qu 4. Can you tell me your views on the following aspects of the WCQ Programme:

a. What did you think of the sessions conducted in a group format with other women?

b. How did you find the delivery of the programme by CF? (Use names)
c. Did you receive support of CF during one-to-one time (e.g., face-to-face, support in the meetings, Telephone calls, or texts)
d. What did you think of the Session content (e.g., setting a quit date, increasing your motivation to quit?)
e. Is there anything missing from the Programme that we need to include in future?
f. If we were to reduce/cut the programme down to its most essential parts, what do you think we would need to 1. Keep and 2. What could we cut?
g. Did you enjoy the flexibility to influence content of the last 6 weeks of Programme?
h. How did you find the access to free NRT, any difficulties accessing NRT at GP or pharmacy?
i. Did you feel you got support from the community pharmacist? How did that make you feel?
j. What did you think of a12-week duration of Programme and its Location?
k. Did you find there was Flexibility to talk about life in general besides smoking? Can you tell me more about that? How did you feel about being able to discuss life in general besides smoking? *(e.g., relationships, children, home life, financial concerns, security of housing situation etc)*
l. Why do you think the course was women only what did you think of that,? What are if any the benefits or drawbacks to this approach? Would you have considered a mixed group support session?

(Qu 3 Questionnaire: Have you cut down since starting the programme?)
If participant has not quit
Qu 5: So from your questionnaire you did (last week) you told me you have not quit smoking yet, is there anything that we could change in the future Programmes that could have helped you more?

Relapse: **(Qu 4 Questionnaire: If you stopped smoking and relapsed, how long did you stop for? What was the main reason for your relapse? What helped you get back on track?)**
On your questionnaire you ticked you relapsed.
Could you tell me more about this?
How did you feel telling the CF or Group about your relapse?
(I was embarrassed in front of the others that I haven't quit yet)

Research trial processes questions:
Qu. 17, 18, 19, 20, 21 Questionnaire
(How satisfied were you with the following: Registration process / Consent process / Support received from facilitators/ Length of the programme/ Follow-up process: Ans: Very satisfied, Satisfied, Neither satisfied nor dissatisfied, Dissatisfied, Very dissatisfied).
(If participant has marked dissatisfied or very dissatisfied explore further)

Qu 6: I can see from you questionnaire you ticked (dissatisfied/very dissatisfied) what was it about the 'process' that made you mark it this way?

Qu 7: How was your experience with the collection of data by the team?
For example, the online registration form, having to meet for signing consent and carrying out questionnaires at the beginning, and again after the 12 weeks.

- What did you think about the paper work?

- And thinking back to the start when you signed up, what did you think of the participant information sheet? *(prompt detailed*)

Qu 8: Was there a long time from your initial interest until the programme began? Was this an issue for you? *loss of motivation?*

Qu 9: What did you think about having your breathing tested/carbon monoxide (CO) reading done during appointments?
- Was it helpful to know your CO score?
- Did you understand why we were interested in your CO score?
- Were you surprised at your score the first time it was taken?
- Did you find the process of providing your CO level comfortable? (*Or a bit intrusive?)*

(Qu 22 Questionnaire: How comfortable were you with your saliva sample being taken?)
Qu 10: What did you think about having your saliva sample taken?
- How did you find the experience?

Qu 11: Do you have any fears or concerns about stopping the programme?
- Will you keeping in touch with other people in the group?
- Continuing to look after your health etc.

Final question:
Qu 16: Is there anything else that you would like to say that you haven't had a chance to tell us about? **(important to ask from the participants point of view, to wrap up the interview)**

Note to researcher - Wrap up
Thank participant for their time and willingness to participate, close focus group
Turn off recorder

2- <u>WCQ2 Community Facilitators</u>

Note to researcher - Before focus group begins:
- Explain the nature of the focus group to participants (voluntary, free to stop taking part at any time, research questions, why their perceptions/opinions are important, what the data will be used for)
- Housekeeping:
  - Recording, transcribed word for word
  - Phones on silent
  - Only one voice at a time, everyone opportunity to speak
  - Provide Participant Information Sheet and Consent form (e.g., confidentiality, anonymity – changing of names)
- Ask participants to sign the consent form, which will be countersigned by the researcher, retain consent forms
- Thank participants for agreeing to take part

- Start recorder

Programme specific questions:
Qu 1. Can you tell me about your experience of being a Community Facilitator on the WCQ Programme?
Note to researcher: For topics not covered in response to above, ask the following questions:

- Why did you get involved in the first place?
- Can you tell me about the training provided by ICS?
- How you see your role as a CF,
- Can you tell me what it is like having a CF ‘partner’ to work with in delivering the sessions?
- What is your opinion of the Flexibility in session content in last 6 sessions of Programme?
- What did you think of the Location/times of sessions?
- How did you find covering core session content ?
- Did you find Challenges of encouraging and motivating women who are struggling to quit/stay quit?

Qu 2. What are 3 things about the WCQ Programme that you think works the best to help women stop smoking*?*
(Prompts if needed):

- meeting other smokers who want to quit,
- the social support of other women in the group,
- having the sessions close to their home,
- the ‘peer-to’peer’ Community Facilitator role,
- free NRT, setting a quit date, ‘passport to quit’,
- help with increasing and maintaining motivation to quit/stay quit, feedback on CO monitoring)

Qu 3. What are 3 things about the WCQ Programme that you think doesn’t work as well/needs more development?
(Prompts if needed):

- covering all of the core session content in the time allowed,
- making sure that no one individual dominates the group
- confidentiality of group discussions,
- 12 week duration too short/too long,
- maintaining motivation of women who are struggling to quit
- how did you find facilitating group conversations that are outside the topic of smoking e.g., home life, relationships, children, security of housing situation, financial pressures etc)(CF- skills/confidence)

Qu 4. Is there anything missing from the WCQ Programme that we need to include in future?

Qu 5. If we were to reduce/cut the programme down to its most essential parts, what do you think we would need to 1. Keep and 2. What could we cut?

Research trial process questions:

Qu 6. Did the fact that the Programme was going to be researched/evaluated make you 'think twice' about being a CF this time around?

Qu 7. The recruitment process was different to recruiting to a standard WCQ Programme as the women were being recruited into a trial. Do you think that this made a difference to the recruitment process? And if so how?
(Prompts if needed):
- there was 50/50 chance to receive WCQ Programme or HSE Programme
- some women found that being part of a research trial off-putting,
- some women found that fact it was 'Trinity' off putting

Qu 8. Due to the trial design areas were randomised to receive the WCQ Programme of the HSE smoking cessation Programme. This meant that the randomisation of areas happened quite close to the delivery of the Programme starting. What impact, if any, did this have on you?
(Prompts if needed):
- Not knowing which area would get the WCQ Programme,
- State of 'readiness' to deliver the Programme

Qu 9. Did you do anything differently in this WCQ Programme to try and encourage the women to continue to attend the Programme?
(Prompts if needed):
- extra persuasion used, additional phone calls, texts etc

Qu 10. We asked you to engage with additional paperwork (e.g., changes to the attendance record, checklist of delivery of session content), how did you find this process?
(Prompts if needed):
- burdensome, unclear who to submit paperwork to
- didn't understand the need/point of some of the information being asked for

Qu 11. Do you feel that you got enough support from the Research Team throughout trial phase?

Qu 12. Is there anything that we could do differently in a future trial to make it easier for CF and the women taking part?

Qu 13. For If we were to sit in or record a session for the research team to evaluate the program?

Final question:
Qu 14. Is there anything else that you would like to say that you haven't had a chance to tell us about?

Note to researcher - Wrap up.
Thank participants for their time and willingness to participate, close focus group. Turn off recorder.
